# Prevalent Per- and Polyfluoroalkyl Substances (PFASs) Pollution in Freshwater Basins in China: A Short Review

**DOI:** 10.3390/toxics13020135

**Published:** 2025-02-13

**Authors:** Jingjing Zhang, Jiaoqin Liu, Riya Jin, Yina Qiao, Jipeng Mao, Zunyao Wang

**Affiliations:** 1 School of Environment and Safety Engineering, North University of China, Taiyuan 030051, China; 15340804892@163.com (J.Z.); qiaoyina203@163.com (Y.Q.); maojipeng2024@126.com (J.M.); 2 State Key Laboratory of Pollution Control and Resources Reuse, School of the Environment, Nanjing University, Naning 210023, China; wangzy@nju.edu.cn

**Keywords:** Per- and Polyfluoroalkyl Substances, bibliometric analysis, presence concentration, surface water, sediment, aquatic organism

## Abstract

Organic pollutants like per- and polyfluoroalkyl substances (PFASs) exhibit persistence, bioaccumulation, resistance to degradation, and high toxicity, garnering significant attention from scholars worldwide. To better address and mitigate the environmental risks posed by PFASs, this paper employs bibliometric analysis to examine the literature on PFASs’ concentrations collected in the Web of Science (WoS) database between 2019 and 2024. The results show that the overall trend of PFASs’ pollution research is relatively stable and increasing. In addition, this study also summarizes the pollution status of traditional PFASs across different environmental media in typical freshwater basins. It analyzes PFASs’ concentrations in surface water, sediment, and aquatic organisms, elucidating their distribution characteristics and potential sources. While perfluorooctanoic acid (PFOA) and perfluorooctane sulfonic acid (PFOS) levels in water environments are declining annually, short-chain PFASs and their substitutes are emerging as primary pollutants. Short-chain PFASs are frequently detected in surface water, whereas long-chain PFASs tend to accumulate in sediments. In aquatic organisms, PFASs are more likely to concentrate in protein-rich organs and tissues. The environmental presence of PFASs is largely influenced by human activities, such as metal plating, fluoride industry development, and industrial wastewater discharge. Currently, the development of PFASs in China faces a complex dilemma, entangled by policy and legal constraints, industrial production demands, the production and use of new alternatives, and their regulation and restriction, creating a vicious cycle. Breaking this deadlock necessitates continuous and active scientific research on PFASs, particularly PFOS, with an emphasis on detailed investigations of environmental sources and sinks. Furthermore, ecological and health risk assessments were conducted using Risk Quotient (RQ) and Hazard Quotient (HQ) methods. Comprehensive comparison indicates that PFASs (such as PFOA) in the majority of freshwater basins are at a low-risk level (RQ < 0.1 or HQ < 0.2), PFOS in some freshwater basins is at a medium-risk level (0.1 < RQ < 1), and no freshwater basin is at a high-risk level. The adsorption and removal approaches of PFASs were also analyzed, revealing that the combination of multiple treatment technologies as a novel integrated treatment technology holds excellent prospects for the removal of PFASs.

## 1. Introduction

Per- and polyfluoroalkyl substances (PFASs) are compounds in which hydrogen atoms in alkane molecules are partially or completely replaced by fluorine atoms [1]. This substitution results in numerous carbon–fluorine (C–F) covalent bonds, imparting exceptional structural stability and resistance to photolysis and hydrolysis. These properties render PFASs difficult to degrade through conventional physicochemical methods. Consequently, PFASs are often termed “forever chemicals” [2]. The fluorine atoms enhance the surface activity, stability, hydrophobicity, and oleophobicity of the perfluoroalkyl fraction [3,4], allowing PFASs to persist in the natural environment [5]. Since the 1950s, PFASs have been integral to various consumer and industrial products [6], including aerospace materials, food contact materials, firefighting foams, household products, semiconductors, and textiles [7,8]. Throughout their life cycle, these products release PFASs, predominantly into aquatic environments, where they are absorbed by biota over time. Beyond bioaccumulation in aquatic and terrestrial species, PFASs also enter the human body via the food web, posing significant health risks [9].

First produced by the 3M Company (Saint Paul, MN, USA) in the 1940s and 1950s, PFASs have been detected in humans, demonstrating potential toxic effects from long-term exposure [10]. Since 2000, major manufacturers in developed countries have phased out the production of perfluorooctane sulfonic acid (PFOS), perfluorooctanoic acid (PFOA), and their salts [3]. However, production has shifted to developing countries, particularly China [11], which had become the largest producer and supplier of PFOS and PFOA by the early 21st century [12]. PFOS and PFOA, the most extensively used PFASs, were listed under the Stockholm Convention on Persistent Organic Pollutants (POPs) in 2009 and 2019, respectively, leading to restrictions or bans on their production, use, and the production of their precursors [13,14]. In 2022, China enacted the “Sanitary Standard for Drinking Water” (GB 5749-2022), which caps the levels of PFOS and PFOA in drinking water at 40 ng/L and 80 ng/L, respectively [15]. In the same year, perfluorohexanesulfonic acid (PFHxS), its salts, and related compounds were added to Annex A of the Stockholm Convention in 2022 [16].

Studies have demonstrated that per- and polyfluoroalkyl substances (PFASs) exhibit long-term stability in aquatic environments [17]. These compounds are not only pervasive in individual media such as surface water [18], sediment [19], and aquatic organisms [20], but also extensively distributed in more complex multi-media environments [21,22,23]. While the literature on per- and polyfluoroalkyl substances (PFASs) is extensive [24,25,26], it predominantly focuses on single environmental media, particularly surface water [27,28,29]. In contrast, comprehensive studies addressing PFASs across multiple media remain relatively limited. To comprehensively understand PFAS pollution in freshwater environments, this study employed bibliometric analysis to examine the publication trends of PFAS-related research and systematically evaluated the concentration levels, composition characteristics, and potential sources of PFASs across various media in typical freshwater basins in recent years. This analysis aimed to provide a valuable reference for future research and management of PFASs in aquatic environments. Table 1 lists the PFASs discussed in this text.

## 2. Data Source and Analysis

### 2.1. Analytical Method

In this study, Origin 2023 was used to generate histograms of the number of published documents per year, thereby analyzing the overall publication trends. Vosviewer (version 1.6.20) was employed for literature visualization analysis. Keywords were visualized in both network and density maps using Vosviewer, and a keyword list was derived to identify the research hotspots both domestically and internationally.

### 2.2. Data Collection

The Web of Science (WoS), as a highly respected database of high-quality digital literature resources, has received widespread recognition among the majority of researchers and has become the tool of choice for bibliometric analyses. To comprehensively reflect the research progress of domestic and foreign scholars in the field of perfluorinated compounds pollution in water environment, this study specifically selected the Web of Science core collection database as the source of original data. In the field of science and technology, the dynamic change in the number of publications of literature is not only a direct reflection of the activity academic research, but also a sensitive indicator of the rise and fall of the corresponding disciplines. Through in-depth analysis of these publication data, we can gain insights into the latest trends and future directions of research on perfluorinated compounds pollution in the water environment.

The Chinese literature was derived from the China National Knowledge Infrastructure Database (CNKI), and the advanced search method was SU% = (“PFASs” + “surface water” + “sediment” + “aquatic organisms”). The English literature is derived from the WoS core database, which records the most representative articles. In the process of data collection, “PFASs”, “presence concentration”, “surface water”, “sediment” and “aquatic organisms” were used as keywords. The data collection cycle was limited to the past 5 years of the data screening phase.

**Figure 1:**
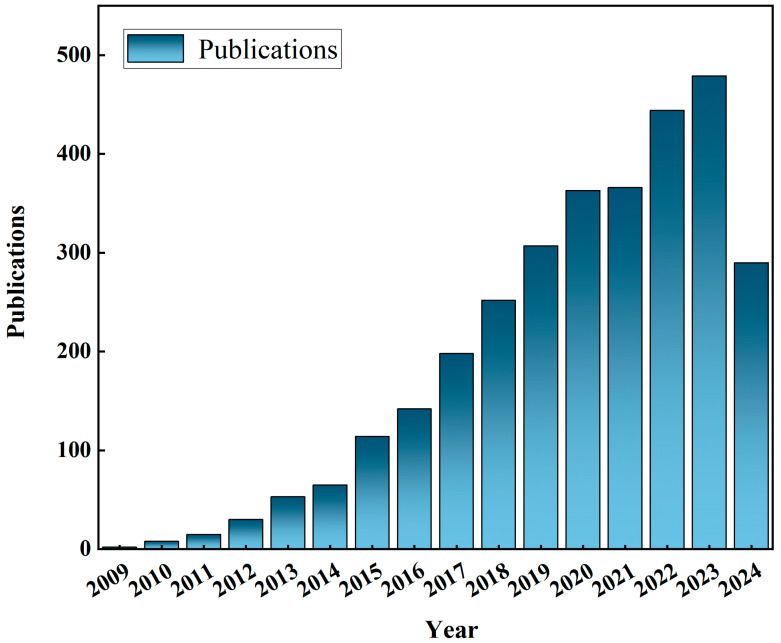
WOS Annual publication on PFASs.

Figure 1 depicts the number of published documents in English, which are as follows. From 2009 to 2023, the literature related to perfluorinated compounds exhibited a steady growth trend overall, from only 2 articles in 2009 to more than 50 articles in 2013, reaching a peak of 479 articles in 2023, and the number of literature citations increased rapidly between 2021 and 2024, indicating that global attention to perfluorinated compounds is increasing.

### 2.3. Research Hotspot

**Figure 2:**
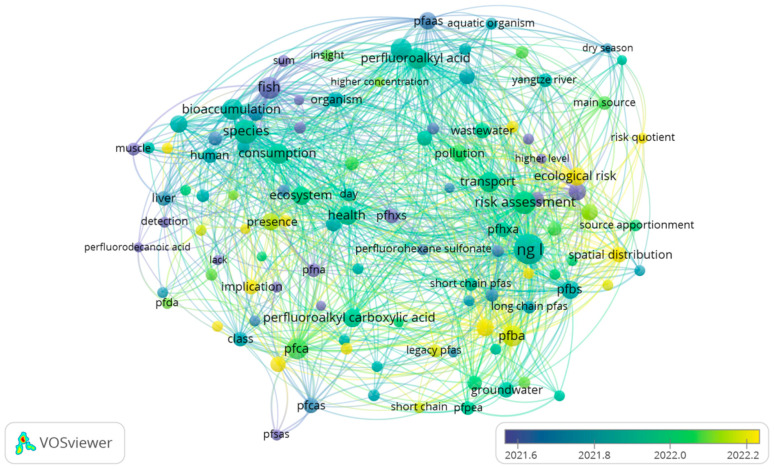
WOS keyword network map.

To further analyze the research content focusing on perfluorinated compounds and their impact on water environmental pollution in recent years, we conducted temporal superposition cluster analysis on keywords extracted from literature samples to predict the research frontier and future trend in this field. Keywords that appeared more than 10 times in both databases were selected for the analysis, and different circular nodes were used to represent different keywords. Node size was correlated with keyword occurrence frequency, and node color represented clustering in different years. The results are shown in Figure 2. The keyword co-occurrence graph shows that the research on perfluorinated compounds in water environmental pollution can be mainly divided into three clusters. These include “surface water”, “sediment”, and “aquatic life”. Based on the trend analysis of the data over the past five years, the research status of perfluorinated compounds in water environments has become a hot spot in the field of environmental science.

## 3. Distribution Characteristics of PFASs in Surface Waters

Surface water serves as the primary carrier of industrial PFASs’ pollution levels and domestic emissions [30], making the collection and analysis of PFASs in surface water essential for understanding their pollution and transfer mechanisms in China. Currently, PFOS and PFOA exhibit high detection frequencies in China’s polluted water bodies, with concentrations generally reaching the maximum ng/L level [31]. This study collected data on PFASs’ concentrations in surface waters of typical domestic freshwater basins, comprehensively summarizing their distribution characteristics and analyzing potential sources of surface water pollutants. The aim was to provide a theoretical basis for potential risk control measures and future research endeavors.

**Figure 3:**
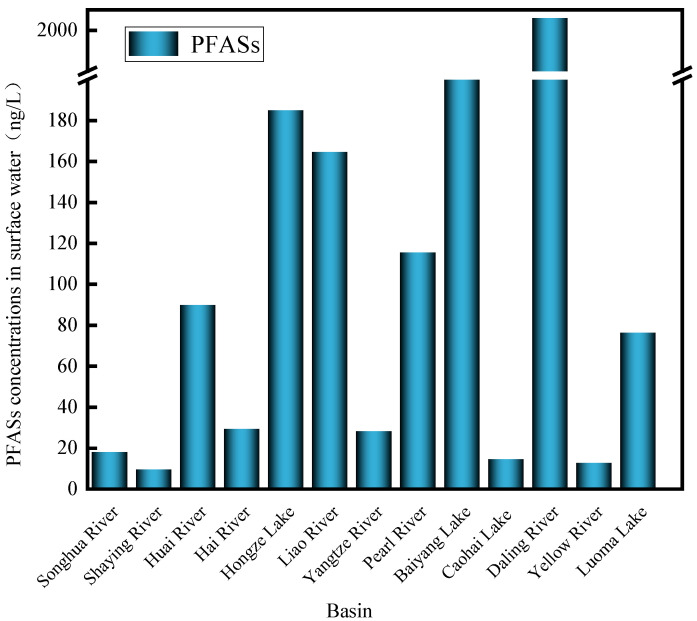
PFAS concentrations in surface water of typical basins in China (ng/L).

China, with its dense river network and numerous lakes, presents a diverse landscape for PFAS pollution. This study summarized and analyzed PFAS concentration ranges (denoted by the average value) in surface waters of typical Chinese basins, as illustrated in Figure 3. The most severely polluted surface water basins were located in East China, followed by Northeast China and North China, with relatively lighter pollution in Southwest and Northwest China, consistent with the findings of Wang et al. [29]. The degree of ∑PFASs’ pollution in typical watercourses was ranked as follows: Daling River (48.4–4578 ng/L, 2005.9 ng/L) [32] > Baiyangdian (140.5–1828.5 ng/L, 880.3 ng/L) [33] > Hongze Lake (63.4–218.0 ng/L, 185.0 ng/L) [34] > Liao River (44.4–781 ng/L, 164.6 ng/L) [35] > Pearl River (11.8–281 ng/L, 115 ng/L) [36] > Huai River (1.59–620 ng/L, 81.7 ng/L) [37] > Luoma Lake (46.09–120.34 ng/L, 76.4 ng/L) [38] > Hai River (1.7–172 ng/L, 26.6 ng/L) [39] > Yangtze River (12.43–77.44 ng/L, 22.53 ng/L) [40] > Songhuajiang River (6.4–32 ng/L, 17 ng/L) [41] > Guizhou Caohai Lake (3.17–16.33 ng/L, 14.4 ng/L) [42] > Yellow River (0.86–90.7 ng/L, 12.6 ng/L) [43] > Shaying River (5.069–20.322 ng/L, 9.4 ng/L) [44]. The highest concentration was found in the Daling River, followed by the Baidianyang River, and the lowest in the Shaying River Basin. Xiaoqing River [45], a tributary of the Yellow River, exhibited an average concentration as high as 28,785.6 ng/L. A 2020 study by Zhang et al. [46] reported that PFAS concentrations in this basin remain very high, at 25,429 ng/L, indicating an urgent need for continuous environmental monitoring and implementation of environmental governance and protection measures in this watershed.

**Figure 4:**
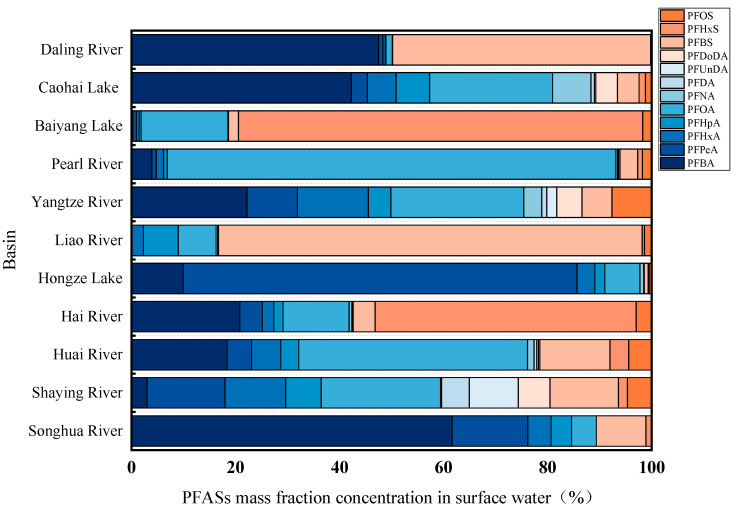
PFASs’ mass fraction in the surface water of typical watershed in China (%).

Figure 4 presented the stacking bar diagram of PFASs in several typical watersheds in China. In the Daling River basin, PFBA and PFBS accounted for 41% and 48% of the mass fractions, respectively, suggesting that these compounds might be the primary substitutes for traditional PFASs in fluorine chemical industry parks. Similar results were observed in the Hongze Lake, Liaohe River, Songhua River, and Caohai Lake basins. Conversely, in the Pearl River and Baidianyang River basins, PFASs such as PFOA and PFOS predominate, indicating that traditional long-chain PFASs are still prevalent in these areas. The data indicated that PFOS has nearly disappeared from the environment, likely due to its classification as a controlled substance in recent years. This classification has led to reduced production and usage, and, consequently, to lower pollution levels and proportions in the environment [45].

The results indicate that the distribution of PFASs in surface water was closely linked to regional industrialization, particularly the direct discharge from fluoropolymer facilities, as the eastern region of China has a well-developed textile and fluoropolymer industry [19]. Although the primary PFASs in the Yangtze River, the Pearl River, and other basins differ, their pollution sources were similar. Direct pollution sources mainly included sewage discharge from fluoridation factories, industrial discharge, and domestic sewage from rubber product manufacturing, fire extinguishers, metal electroplating, textiles, and more. Indirect pollution sources encompass wet and dry deposition [46], surface runoff [47], and the biological or chemical transformation of PFASs precursors in the environment [22], such as 6:2 fluorotelomer sulfonic acid (6:2 FTS) as a substitute for PFOS and PFPeA as one of its transformation products [48].

The comparison and summary of PFASs in surface water across different regions reveal significant variations in the types of detected PFASs. Short-chain PFASs dominate in several freshwater basins, likely because short-chain compounds like PFBA and PFPeA have been used as substitutes for long-chain PFASs, such as PFOA in industrial processes. Short-chain PFASs not only exhibit similar persistence, allowing them to remain stable in the water environment for extended periods, but also possess stronger water solubility, making them more likely to enter the hydrosphere and caused pollution [42]. This trend was observed in basins such as the Songhua River, Haihe River, and Hongze Lake. Additionally, the higher solubility of short-chain PFASs compared to long-chain PFASs contributes to their prevalence [43]. However, areas like the Haihe River Basin and Caohai Lake in Guizhou still show dominance of PFOA and PFOS, possibly due to their extensive past use and resistance to degradation, which continue to impact the environment despite bans and reductions in production and use.

## 4. Distribution Characteristics of PFASs in Sediments

Sediment, as an integral part of the water environment, possesses a high capacity to enrich organic pollutants and plays a crucial role in their migration and transformation. During periodic changes in the water environment, the water solubility of PFASs decreased with increasing carbon chain length, leading to their precipitation onto particles and deposition in sediments [22]. PFASs adsorbed to sediments could migrate and transform under certain conditions, desorbing back into the water environment. Consequently, sediments are considered a primary source of PFASs for benthic food webs in lakes [48].

**Figure 5:**
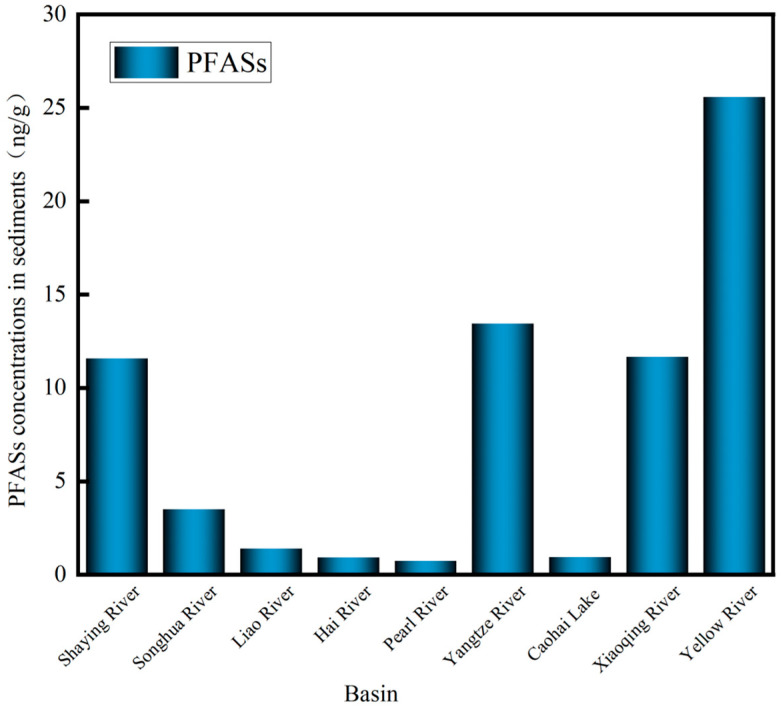
PFAS concentrations in the sediment of typical basins in China (ng/g).

This study summarized the concentration levels of PFASs in sediments across different basins in China (represented by average values), as illustrated in Figure 5 The most severe PFASs pollution in sediments occurred in northern and eastern China, followed by the northeast, with relatively lighter pollution in the north and south. The degree of ∑PFASs’ pollution in typical drainage basins was ranked as follows: Yellow River (15.57–36.42 ng/g, 25.58 ng/g) [43] > Yangtze River (3.83–20.33 ng/g, 15.18 ng/g) [49] > Xiaoqing River (9.42–34.9 ng/g, 11.67 ng/g) [45] > Shaying River (6.46–20.05 ng/g, 11.58 ng/g) > Songhua River (2.0–5.2 ng/g, 3.5 ng/g) [41] > Liao River (0.54–2.34 ng/g, 1.03 ng/g) [35] > Hai River (0.024–2.845 ng/g, 0.927 ng/g) [39] > Caohai Lake of Guizhou Province (0.11–1.34 ng/g, 0.95 ng/g) [42] > Pearl River (0.23–1.44 ng/g, 0.74 ng/g) [36]. The highest concentration of PFASs was found in the Yellow River sediment, followed by the Yangtze River and Pearl River Basin. This was likely due to the presence of tanneries, paper mills, and other heavily polluting enterprises around the Yellow River basin [43]. In the Yangtze River Basin, the main sources of PFASs in sediments were electroplating, fast food packaging, textiles, etc. [50]. The sediment of the Shaying River exhibited medium PFAS levels, potentially linked to ecological factors and the urban water hub project [51]. Basins such as the Songhua River, Liaohe River, Haihe River, Caohai Lake of Guizhou Province, and Pearl River showed lower PFAS concentrations, possibly because these areas are primarily plains located in North and South China [6].

**Figure 6:**
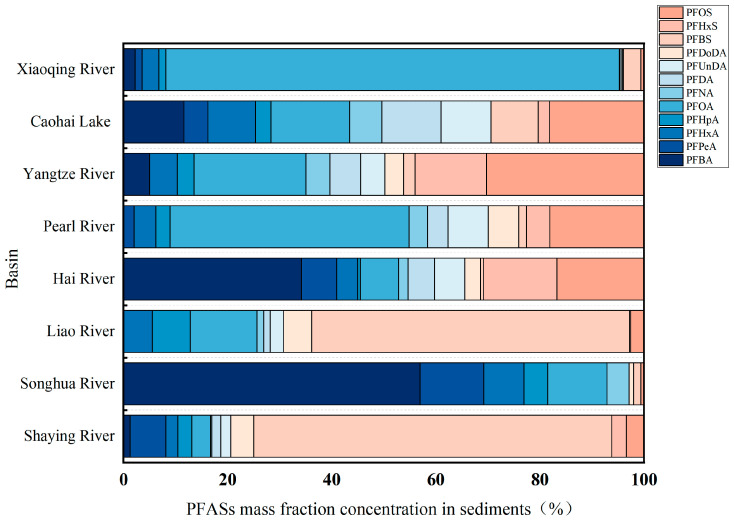
PFASs’ mass fraction in the sediments of typical watersheds in China (%).

As illustrated in Figure 6, the surface sediments of the lower Yangtze River predominantly contain PFOA and PFOS, a distribution similar to that in the Caohai Lake and Xiaoqing River in Guizhou. In the Luoma Lake [38], Songhua River, and Haihe River basins, short-chain PFBA is the dominant PFASs. The Shaying River and Liaohe River exhibited relatively high mass fractions for PFBS, with PFSAs constituting 74.17% of the PFASs in the Shaying River. This could be attributed to the higher hydrophobicity of PFSAs compared to PFCAs due to differences in functional groups [52], which facilitates their attachment to sediments—a finding consistent with previous research [53]. Additionally, the increasing use of short-chain PFASs such as PFBA, PFHxA, and PFBS as substitutes for long-chain substances has led to their rising concentrations in sediments [54]. In some areas, the concentration of short-chain PFASs is higher than that of long-chain PFASs, this phenomenon can be attributed to the combined action of many factors. Firstly, the hydrophobic portion of organic matter adsorbed on sediment surfaces enhances the adsorption of short-chain PFASs [53]. Secondly, previous studies have demonstrated that the adsorption of short-chain PFASs carboxylates in sediments is primarily driven by electrostatic interactions between the carboxylic acid groups and charged particles on the sediment surface [55]. Finally, sediments serve as primary habitats for benthic organisms, and factors such as water pH, salinity [56], sediment composition [57], biotransformation, and bioaccumulation processes also significantly influence the adsorption of short-chain PFASs [58].

**Figure 7:**
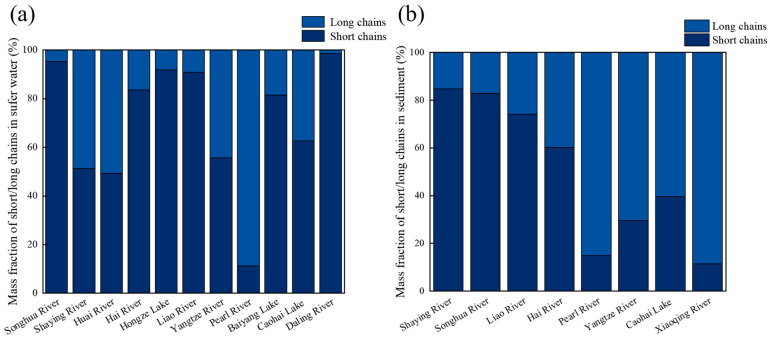
(**a**) Mass fraction of short/long chains in sediment (%) and (**b**) mass fraction of short/long chains in surface water (%).

Comparing PFASs’ concentrations in surface water and sediment reveals significant differences in concentration and composition across these media. In terms of concentration, PFAS levels in surface water in some areas have reached notable levels, whereas sediment concentrations were more uniformly distributed. Figure 7 presents a comparison of short- and long-chain PFASs in surface water and sediments from various regions. The figure reveals that short-chain PFASs dominate in multiple freshwater basins across most regions, whereas long-chain PFASs are predominantly found in sediments. Despite these differences, surface water and sediment, both parts of the hydrosphere, shared similar pollution sources, primarily from the production and emissions of fluorine plants in heavy industrial cities.

## 5. Pollution Status of PFASs in Aquatic Organisms

PFASs enter aquatic environments through various pathways, posing threats to ecosystems. The impact of PFASs on water quality could be assessed using aquatic biological indicators. Fish, shellfish, and benthos, as key aquatic organisms, accumulate PFASs from contaminated sediments and transfer these pollutants through the food web to higher trophic levels, including humans, resulting in adverse health effects [59]. To mitigate the impact of PFASs in aquatic ecosystems and trophic networks, it was crucial to verify their presence in aquatic organisms and study their effects [60]. Understanding the distribution characteristics of PFASs in aquatic organisms is vital for assessing bioaccumulation and potential risks.

Detected levels of PFASs in aquatic organisms vary significantly. Table 2 presents the concentration range of PFASs in aquatic organisms across different regions of China. In the Xiaoqing River [45], ∑PFAS concentrations ranged from 86.1 ng/g to 17,100 ng/g, with gastropods showing the highest levels, followed by bivalves, crustaceans, fish, and plankton. Similar observations were made in the Baidianyang and Taihu Lake basins [22]. This variation could be attributed to (1) differing enrichment abilities, environmental conditions, physiology, and feeding habits of organisms [61]; (2) varying bioaccumulation and biomagnification properties of PFASs with different carbon chain lengths [62]; and (3) differences in geographical locations and regional living environments.

PFAS concentrations differ across various tissues and organs within the same organism. Chen et al. [63] observed that PFAS levels in fish species followed a specific patterns: juvenile carnivorous > omnivorous, and adult carnivorous > omnivorous > herbivorous, which is consistent with Liu Sifan et al. [64]. This pattern was linked to trophic levels, feeding habits, and living habits of fish. In fish from Taihu Lake [22], PFAS concentrations were the highest in the liver, followed by the heart (or spleen), kidney, gills, and muscle, aligning with findings by Chen et al. [65]. Higher ΣPFAS levels in fish liver compared to muscle, which is consistent with studies from the Jiulong River basin [66] and the Yangtze River [67], were attributed to PFASs’ strong affinity for proteins, leading to accumulation in protein-rich tissues and organs like the liver and blood [68].

Analyzing PFAS concentrations in aquatic organisms across different regions showed nearly 100% detection rates for PFOA, PFOS, and long-chain PFASs. Similar findings in previous food web studies [69] indicate that long-chain PFASs were easily amplified by trophic organisms, while short-chain PFASs were more likely to be diluted [70]. The content of PFASs varies significantly among species and within tissues of the same species. The hydrophobic and oleophobic properties of PFASs caused enrichment in protein-rich organs and tissues, forming stable structures [71]. Additionally, the living habits and environment of aquatic organisms influenced PFAS content. For example, high PFAS levels in eels and loach from Baiyangdian Lake were likely due to prolonged contact with sediments [24].

**Table 2:** Concentration range of PFASs in aquatic organisms in different regions of China.

PFASs(ng/g)													
Source	Years	Species	PFBA	PFHpA	PFOA	PFNA	PFDA	PFUnDA	PFDoDA	PFBS	PFHxS	PFOS	References
Xiaoqing River	2022	phytoplankton	n.d.	n.d.	65.81–79.24	0.36–0.48	0.33–0.47	0.41–0.43	0.28–0.34	n.d.	n.d.	0.79–1.87	[45]
		zooplankter	n.d.	n.d.	88.81–156.21	0.33–0.61	0.51–0.75	0.56–0.76	0.35–0.51	n.d.	n.d.	4.13–8.77	
		ostracean	9.25–9.75	n.d.	53.79–57.91	0.34–0.46	-	0.41–0.53	0.24–0.28	n.d.	n.d.	4.16–4.46	
		oyster	16.68–17.64	1.48–1.70	260.04–296.18	0.75–0.87	0.37–0.41	0.25–0.33	0.03–0.13	n.d.	n.d.	3.28–3.74	
		clam	0.00–14.19	n.d.-1.86	0.00–682.43	0.00–6.17	0.00–0.82	0.00–5.23	0.00–0.30	n.d.–2.46	n.d.	0.00–4.87	
		conch	263.49–267.03	n.d.	44.63–47.27	0.36–0.48	0.29–0.35	0.28–0.30	0.21–0.23	n.d.	n.d.	2.61–2.84	
		prawn	4.39–5.79	n.d.–0.71	137.23–165.29	0.90–1.74	1.53–2.40	1.27–1.99	0.42–1.36	n.d.	n.d.	8.75–10.31	
		fish	1.10–16.01	n.d.–3.61	30.83–308.62	0.43–1.29	0.97–3.83	0.68–2.15	0.17–1.07	n.d.–3.27	n.d.	4.89–19.87	
Jiulong River	2022	fish muscle	–	n.d.–2.92	10.83–24.31	n.d.–10.29	0.13–10.31	0.82–11.73	0.45–6.84	n.d.	3.15–25.23	0.92–47.41	[66]
		fish liver	–	n.d.–3.62	13.78–234.67	n.d.–19.38	0.28–82.06	2.42–96.18	1.19–40.0	n.d.–0.77	6.08–82.82	2.64–793.34	
Baiyang Lake	2018	shrimp	–	–	0.17	0.28	0.88	0.45	0.12	–	32.94	6.84	[33]
		crab	–	–	0.39–1.09	0.05–0.38	0.35–0.97	0.21–0.82	0.03–0.23	–	0.47–3.56	1.87–6.63	
		tortoise	–	–	n.d.–0.08	n.d.–0.02	1.11–1.27	1.11–1.27	0.23–0.25	–	0.00–0.02	4.47–4.71	
		loach	–	–	29.60–33.76	0.69–1.03	1.38–1.72	0.87–1.53	0.24–0.52	–	33.59–45.95	13.26–23.68	
Yangtze River	2021	crucian muscle	n.d.–2.45	n.d.–3.00	0.30–0.49	n.d.–2.30	n.d.–4.10	0.60–3.10	n.d.–3.40	n.d.–4.40	–	2.00–10.30	[67]
		crucian liver	n.d.–5.80	n.d.–22.30	2.30–24.60	n.d.–11.50	n.d.–11.00	2.40–16.80	n.d.–11.7	0.40–9.50	n.d.–11.60	12.90–78.70	

n.d. indicates that the compounds were not detected or below the detection limit.

## 6. Risk Evaluation

Each freshwater basin is intricately linked to human health. Ecological and health risk assessments were carried out by Risk Quotient (RQ) [33,41,42] and Hazard Quotient (HQ) [31,41]. Comprehensive comparisons reveal that PFASs (such as PFOA, etc.) in the majority of freshwater basins were at a low-risk level (RQ < 0.1 or HQ < 0.2), PFOS in certain freshwater basins was at a medium-risk level (0.1 < RQ < 1), and no freshwater basins were at a high-risk level. However, none of the studies have taken into account toxicity risk assessment of mixtures of several or more PFASs. Owing to the frequent toxicological interactions among chemicals and the scarcity of mixture risk assessment methods capable of accounting for these interactions, further studies on mixture risks associated with several or more substances have not been carried out. As a significant scientific issue, risk assessment of mixtures demands further research in the future.

## 7. Removal of PFASs in Aqueous Environments

PFASs are a typical persistent organic pollutant detected in various water environments and have attracted widespread attention due to their adverse ecological and human health effects. At present, the most common methods of removing PFASs are adsorption with using adsorbents (such as activated carbon and biochar [25,72,73] and ion exchange resins [74,75,76,77,78]. In recent years, significant progress has been made in optimizing carbonaceous adsorbents, such as the use of biomass as a cost-effective feedstock [79], enhanced magnetic properties to promote separation [80], and increased surface alkalinity to improve performance [81]. For resins, the adsorption rate and capacity of PFAS mainly rely on the polymer matrix, functional groups, and porosity of the specific resin [82]. The mechanism by which resins remove short-chain PFAS is typically attributed to anion exchange, while long-chain PFASs can be further removed through micelle/semi-micelle formation and agglomeration [83]. Advanced approaches, such as ion exchange, fixed-phase adsorption, and high-pressure membrane systems (reverse osmosis and nanofiltration), also hold potential for PFAS removal [26,84,85,86,87]. Glutaraldehyde (GTH) cross-linked chitosan (CTN) biopolymer-based and polyvinylimidazole (PEI) functionalized ((GTH-CTN-PEI)) aerogels have been demonstrated to be promising for removing mixtures of long- and short-chain perfluoroalkyl substances and PFASs from water simultaneously [88]. A number of emerging treatment technologies are gradually emerging, including fluorinated adsorbent [89], metal–organic framework adsorption [90,91] and covalent organic framework adsorption [92], flocculation [93,94,95], photocatalytic oxidation [96] and electrochemical oxidation [97,98,99], as well as natural degradation methods, such as constructed wetlands [100,101].

In a pilot-scale study, foam fractionation was used to concentrate PFAS in the target medium, which was then combined with electrochemical techniques to degrade PFASs, thereby achieving defluorination and reducing water toxicity. Dey et al. [96] pointed out that carbonaceous materials can be ideal candidates for the adsorption and photocatalytic treatment of PFASs-contaminated water. This further highlights the recent development of innovative “concentration and degradation” methods, which selectively adsorb trace concentrations of PFASs onto photoactive surface sites with enhanced catalytic activity. This technology is more energy-efficient than traditional, energy-intensive photocatalysis and represents a promising new approach that combines adsorption and degradation. Rao et al. [102] used a combination of flocculation and UV/S/I treatment to address two types of wastewater containing primarily short-chain PFASs. The PFAS removal rate reached >99% within 20 h, while the maximum defluorination rates for the two types of waste saltwater reached 85% and 70%, respectively. This research advances UV technology for PFAS destruction and enhances the sustainability of ion-exchange resin removal of PFASs. Additionally, Leen et al. [103] indicated that the combination of electro-Fenton and electrochemistry stands out as a promising PFAS-degradation scheme. The system utilizes two processes with similar oxidation mechanisms, eliminating the need for multiple energy sources. Moreover, both processes generate hydrogen peroxide in situ, eliminating the need for external chemical input. Operating in a cathode–anode cycle, the system exhibits a short residence time and a high removal rate, indicating great potential for direct scale-up in large treatment plants.

The integration of multiple technologies is designed to provide a comprehensive PFAS removal method that ultimately improves efficiency and effectiveness. As a novel comprehensive treatment technology, the combination of multiple treatment techniques holds excellent prospects for the removal of PFASs.

## 8. Conclusions and Perspectives

(a) This study summarized the concentrations of PFASs in surface water, sediments, and aquatic organisms in typical freshwater basins in China, detailing their distribution characteristics and potential sources. The results indicated that since PFOA and PFOS were listed in the Stockholm Convention on Persistent Organic Pollutants, their manufacturing, use, and disposal have been effectively controlled, resulting in decreasing levels of PFOA and PFOS in the water environment.
(b) The distribution behavior of various PFASs in different water media showed significant variation. The most severe surface water pollution was observed in East China, followed by Northeast and North China, with short-chain PFASs showing higher detection rates in surface water. Sediment pollution was the most severe in the east and north, followed by the northeast, with relatively lighter pollution in North and South China. Long-chain PFASs tended to accumulate more in sediments. Aquatic organisms exhibit nearly 100% detection rates for long-chain PFASs, as longer carbon chains facilitate greater accumulation in organisms. Higher PFAS concentrations were found in tissues and organs such as the liver.
(c) The development of PFASs in China is currently entangled in a cycle of policy and legal restrictions, industrial production demand, the creation and use of new substitutes, and subsequent regulation and restriction of these substitutes. Continuous and proactive scientific research on PFASs, particularly on environmental sources and sinks, is essential. A transition to safer alternatives should be promoted to mitigate the impact of PFASs on human health and the environment.
(d) Through single-substance risk assessment, a comprehensive comparison revealed that PFASs, in the majority of freshwater basins, were at a low-risk level, PFOS in some freshwater basins was at a moderate-risk level, and no freshwater basin was at a high-risk level. There is an urgent requirement for a hybrid risk assessment approach to comprehensively evaluate the risk level of a basin by integrating multiple substances.
(e) Single methods for removing PFASs have distinct limitations, and the combination of multiple treatment technologies, as new, integrated treatment technologies hold outstanding prospects for the removal of PFASs.

## Figures and Tables

**Table 1:** PFASs names and abbreviations in this work.

Name	Abbreviation	Molecular Formula
Perfluorobutyric acid	PFBA	C_3_F_7_COOH
Perfluoron pentanoic acid	PFPeA	C_4_F_9_COOH
Perfluorohexanoic acid	PFHxA	C_5_F_11_COOH
Perfluoronheptanoic acid	PFHpA	C_6_F_13_COOH
Perfluorooctanoate acid	PFOA	C_7_F_15_COOH
Perfluorononanoic acid	PFNA	C_8_F_17_COOH
Perfluorodecaoic acid	PFDA	C_9_F_19_COOH
Perfluoroundecanoic acid	PFUnDA	C_10_F_21_COOH
Tricosafluorododecanoic acid	PFDoDA	C_11_F_23_COOH
Perfluorobutanesulfonic acid	PFBS	C_4_HF_9_O_3_S
Perfluorohexanesulfonic acid	PFHxS	C_6_HF_13_O_3_S
Perfluorooctanesulfonic acid	PFOS	C_8_HF_17_O_3_S

## Data Availability

The data are contained within the article.

## References

[1] Pan Y., Zhang H., Cui Q., Sheng N., Yeung L.W.Y., Sun Y., Guo Y., Dai J. (2018). Worldwide Distribution of Novel Perfluoroether Carboxylic and Sulfonic Acids in Surface Water. Environ. Sci. Technol..

[2] Brunn H., Arnold G., Körner W., Rippen G., Steinhäuser K.G., Valentin I. (2023). PFAS: Forever chemicals—Persistent, bioaccumulative and mobile. Reviewing the status and the need for their phase out and remediation of contaminated sites. Environ. Sci. Eur..

[3] Buck R.C., Franklin J., Berger U., Conder L.T., Voogt P.d., Jensen A.A., Kannan K., Mabury S.J., Leeuwen S.P.J. (2011). Perfluoroalkyl and polyfluoroalkyl substances in the environment: Terminology, classification, and origins. Integr. Environ. Assess. Manag..

[4] Brendel S., Fetter É., Staude C., Vierke L., Biegel-Engler A. (2018). Short-chain perfluoroalkyl acids: Environmental concerns and a regulatory strategy under REACH. Environ. Sci. Eur..

[5] Gar Alalm M., Boffito D.C. (2022). Mechanisms and pathways of PFAS degradation by advanced oxidation and reduction processes: A critical review. Chem. Eng. J..

[6] Christensen K.Y., Raymond M., Meiman J. (2019). Perfluoroalkyl substances and metabolic syndrome. Int. J. Hyg. Environ. Health.

[7] Langberg H.A., Breedveld G.D., Slinde G.A., Grønning H.M., Høisæter Å., Jartun M., Rundberget T., Jenssen B.M., Hale S.E. (2020). Fluorinated Precursor Compounds in Sediments as a Source of Perfluorinated Alkyl Acids (PFAA) to Biota. Environ. Sci. Technol..

[8] Pan C., Wang Y., Yu K., Zhang W., Zhang J., Guo J. (2020). Occurrence and distribution of perfluoroalkyl substances in surface riverine and coastal sediments from the Beibu Gulf, south China. Mar. Pollut. Bull..

[9] Evich M.G., Davis M.J.B., McCord J.P., Acrey B., Awkerman J.A., Knappe D.R.U., Lindstrom A.B., Speth T.F., Tebes-Stevens C., Strynar M.J. (2022). Per- and polyfluoroalkyl substances in the environment. Science.

[10] Lindstrom A.B., Strynar M.J., Libelo E.L. (2011). Polyfluorinated Compounds: Past, Present, and Future. Environ. Sci. Technol..

[11] Song X., Vestergren R., Shi Y., Huang J., Cai Y.Q. (2018). Emissions, Transport, and Fate of Emerging Per- and Polyfluoroalkyl Substances from One of the Major Fluoropolymer Manufacturing Facilities in China. Environ. Sci. Technol..

[12] Wang Q., Ruan Y., Lin H., Lam P.K.S. (2020). Review on perfluoroalkyl and polyfluoroalkyl substances (PFASs) in the Chinese atmospheric environment. Sci. Total Environ..

[13] (2009). UNEP Report of the Conference of the Parties of the Stockholm Convention on Persistent Organic Pollutants on the Work of Its Fourth Meeting. http://chm.pops.int/portals/0/repository/cop4/unep-pops-cop.4-38.english.pdf.

[14] (2017). UNEP Report of the Conference of the Parties to the Stockholm Convention on Persistent Organic Pollutants on the Work of Its Eighth Meeting. https://www.pops.int/TheConvention/ConferenceoftheParties/Meetings/COP8/tabid/5309/Default.aspx.

[15] Hu J., Zhang Z., Yang M. (2023). Study on setting the standard limits of perfluorooctanoic acid and perfluorooctane sulfonic acid in Drinking Water Hygienic Standard (GB5749-2022). Chin. J. Prev. Med..

[16] U.E.P.A. (2022). The New POPs Under the Stockholm Convention.

[17] Remucal C.K. (2019). Spatial and temporal variability of perfluoroalkyl substances in the Laurentian Great Lakes. Environ. Sci. Process. Impacts.

[18] Chen R., Li G., Yu Y., Ma X., Zhuang Y., Tao H., Shi B.Y. (2019). Occurrence and transport behaviors of perfluoroalkyl acids in drinking water distribution systems. Sci. Total Environ..

[19] Dong W., Liu B., Song Y., Zhang H., Li J.Y., Cui X.Y. (2018). Occurrence and Partition of Perfluorinated Compounds (PFCs) in Water and Sediment from the Songhua River, China. Arch. Environ. Contam. Toxicol..

[20] Li F., Huang H., Xu Z., Ni H., Yan H., Chen R., Luo Y., Pan W., Long J.Y., Ye X.F. (2017). Investigation of Perfluoroalkyl Substances (PFASs) in Sediments from the Urban Lakes of Anqing City, Anhui Province. China Bull. Environ. Contam. Toxicol..

[21] Lin X., Wang S., Li Q., Li Y.Y., Yamazaki E., Yamashita N., Wang X.H. (2021). Occurrence, partitioning behavior and risk assessments of per- and polyfluoroalkyl substances in water, sediment and biota from the Dongshan Bay, China. Chemosphere.

[22] Liang X., Zhou J., Yang X., Jiao W.Q., Zhu L.Y., Yang X. (2023). Disclosing the bioaccumulation and biomagnification behaviors of emerging per/polyfluoroalkyl substances in aquatic food web based on field investigation and model simulation. J. Hazard. Mater..

[23] Diao J., Chen Z., Wang T., Su C.S., Sun Q.P., Guo Y.J., Zheng Z., Wang L., Li P., Liu W.H. (2022). Perfluoroalkyl substances in marine food webs from South China Sea: Trophic transfer and human exposure implication. J. Hazard. Mater..

[24] Tan H., Pan C., Yin C., Yu K.F. (2023). Toward systematic understanding of adsorptive removal of legacy and emerging per-and polyfluoroalkyl substances (PFASs) by various activated carbons (ACs). Environ. Res..

[25] Md N., Jiang T., Liang Y. (2024). Structure and mechanism of nanoengineered membranes toward per- and polyfluoroalkyl sub-stances (PFAS) removal from water: A critical review. J. Water Process Eng..

[26] Makowska K., Martín J., Rychlik A., Aparicio I., Santos J.L., Alonso E., Gonkowski S. (2021). Assessment of exposure to perfluoroalkyl substances (PFASs) in dogs by fur analysis. Environ. Pollut..

[27] Huang L., Wang W., Zhang Y. (2024). Research progress on the pollution status of per-and polyfluoroalkyl substances (PFASs) in surface water: A review. Environ. Chem..

[28] Zhang L., Wang M., Zhang M., Yang D.J. (2023). Per- and polyfluoroalkyl substances in Chinese surface waters: A review. Ecotoxicol. Environ. Saf..

[29] Wang J., Shen C., Zhang J., Lou G.Y., Shan S.D., Zhao Y.Q., Man Y.B., Li Y.L. (2024). Per- and polyfluoroalkyl substances (PFASs) in Chinese surface water: Temporal trends and geographical distribution. Sci. Total Environ..

[30] Wang F., Zhuang Y., Dong B., Wu J. (2022). Review on Per- and Poly-Fluoroalkyl Substances’ (PFASs’) Pollution Characteristics and Possible Sources in Surface Water and Precipitation of China. Water.

[31] Zhu Y., Tang J., Li M. (2021). Contamination status of perfluorinated compounds and its combined effects with organic pollutants. Asian J. Ecotoxicol..

[32] Gao L., Liu J., Bao K., Chen N., Meng B. (2020). Multicompartment occurrence and partitioning of alternative and legacy per- and polyfluoroalkyl substances in an impacted river in China. Sci. Total Environ..

[33] Guo R., Liu X., Liu J., Liu Y., Qiao X., Ma M., Zheng B., Zhao X. (2020). Occurrence, partition and environmental risk assessment of per- and polyfluoroalkyl substances in water and sediment from the Baiyangdian Lake, China. Sci. Rep..

[34] Huang J., Tao Y., Huang T. (2023). Occurrence, Sources and Health Risk Assessment of Per- and Polyfluoroalkyl Substances in Sur-face Water of Hongze Lake. Res. Environ. Sci..

[35] Chen X., Zhu L., Pan X., Fang S., Zhang Y., Yang L. (2015). Isomeric specific partitioning behaviors of perfluoroalkyl substances in water dissolved phase, suspended particulate matters and sediments in Liao River Basin and Taihu Lake, China. Water Res..

[36] Li W., Li H., Zhang D., Tong Y., Li F., Cheng F., Huang Z., You J. (2022). Legacy and Emerging Per- and Polyfluoroalkyl Substances Behave Distinctly in Spatial Distribution and Multimedia Partitioning: A Case Study in the Pearl River, China. Environ. Sci. Technol..

[37] Du D., Lu Y., Zhou Y., Zhang M., Wang C., Yu M., Song S., Cui H., Chen C. (2022). Perfluoroalkyl acids (PFAAs) in water along the entire coastal line of China: Spatial distribution, mass loadings, and worldwide comparisons. Environ. Int..

[38] Huang J., Wu W., Huang T. (2022). Characteristics, Sources, and Risk Assessment of Perlyfluoroalkyl Substances in Surface Water and Sediment of Luoma Lake. Environ. Sci..

[39] Li Y., Feng X., Zhou J., Zhu L. (2020). Occurrence and source apportionment of novel and legacy poly/perfluoroalkyl substances in Hai River basin in China using receptor models and isomeric fingerprints. Water Res..

[40] Leng Y., Xiao H., Li Z., Liu Y., Huang K., Wang J. (2021). Occurrence and ecotoxicological risk assessment of perfluoroalkyl substances in water of lakes along the middle reach of Yangtze River, China. Sci. Total Environ..

[41] Zhang X., Hu T., Yang L., Guo Z. (2018). The Investigation of Perfluoroalkyl Substances in Seasonal Freeze–Thaw Rivers During Spring Flood Period: A Case Study in Songhua River and Yalu River, China. Bull. Environ. Contam. Toxicol..

[42] Zeng S., Yang H., Peng J. (2021). Pollution characteristics and risk assessment of perfluorinated compounds in surface water and sediments of Caohai Lake of Guizhou Province. Environ. Chem..

[43] Zhou J., Li Z., Guo X., Li Y., Wu Z., Zhu L. (2019). Evidences for replacing legacy per- and polyfluoroalkyl substances with emerging ones in Fen and Wei River basins in central and western China. J. Hazard. Mater..

[44] Zhang Y.-H., Ding T.-T., Huang Z.-Y., Liang H.-Y., Du S.-L., Zhang J., Li H.-X. (2023). Environmental exposure and ecological risk of perfluorinated substances (PFASs) in the Shaying River Basin, China. Chemosphere.

[45] Li Y., Yao J., Zhang J., Pan Y., Dai J., Ji C., Tang J. (2021). First Report on the Bioaccumulation and Trophic Transfer of Perfluoroalkyl Ether Carboxylic Acids in Estuarine Food Web. Environ. Sci. Technol..

[46] Huang K., Li Y., Bu D., Fu J., Wang M., Zhou W., Gu L., Fu Y., Cong Z., Hu B. (2022). Trophic Magnification of Short-Chain Per- and Polyfluoroalkyl Substances in a Terrestrial Food Chain from the Tibetan Plateau. Environ. Sci. Technol. Lett..

[47] Zhao P., Xia X., Dong J., Xia N., Jiang X., Li Y., Zhu Y. (2016). Short- and long-chain perfluoroalkyl substances in the water, suspended particulate matter, and surface sediment of a turbid river. Sci. Total Environ..

[48] Lescord G.L., Kidd K.A., De Silva A.O., Williamson M., Spencer C., Wang X.W., Muir D.C.G. (2015). Perfluorinated and polyfluorinated compounds in lake food webs from the Canadian high Arctic. Environ. Sci. Technol..

[49] Hua Z., Yu L., Liu X., Zhang Y., Ma Y.X., Lu Y., Wang Y.F., Yang Y.D., Xue H.Q. (2021). Perfluoroalkyl acids in surface sediments from the lower Yangtze River: Occurrence, distribu-tion, sources, inventory, and risk assessment. Sci. Total Environ..

[50] Li T., Chen Y., Wang Y., Tan Y., Jiang C.X., Yang Y.Y., Zhang Z.L. (2024). Occurrence, source apportionment and risk assessment of perfluorinated compounds in sed-iments from the longest river in Asia. J. Hazard. Mater..

[51] Zhang X., Duan B., He S., Lu Y. (2022). Simulation study on the impact of ecological water replenishment on reservoir water environment based on Mike21—Taking Baiguishan reservoir as an example. Ecol. Indic..

[52] Li J., Ai Y., Hu J., Xu N., Song R., Zhu Y.R., Sun W.L., Ni J.R. (2020). Polyfluoroalkyl substances in Danjiangkou Reservoir, China: Occurrence, composition, and source appointment. Sci. Total Environ..

[53] Chen H., Reinhard M., Yin T., Nguyen T.V., Tran N.H., Gin K.Y.H. (2019). Multicompartment distribution of perfluoroalkyl and polyfluoroalkyl substances (PFASs) in an urban catchment system. Water Res..

[54] Li F., Duan J., Tian S., Ji H., Zhu Y., Wei Z., Zhao D. (2020). Short-chain per- and polyfluoroalkyl substances in aquatic systems: Occurrence, impacts and treatment. Chem. Eng. J..

[55] Munoz M., Budzinski H., Labadie P. (2017). Influence of environmental factors on the fate of legacy and emerging per- and polyfluoroalkyl substances along the salinity/turbidity gradient of a macrotidal estuary. Environ. Sci. Technol..

[56] Pan G., Jia C., Zhao D., You C., Chen H., Jiang G.B. (2009). Effect of cationic and anionic surfactants on the sorption and desorption of perfluorooctane sulfonate (PFOS) on natural sediments. Environ. Pollut..

[57] Luo X., Yang Z., He M. (2005). Sorption of hydrophobic organic contaminants by natural organic matter in soils and sedi-ments. Soils.

[58] Nakata H., Kannan K., Nasu T., Hyeon-Seo C., Ewan S., Akira T. (2006). Perfluorinated contaminants in sediments and aquatic organisms collected from shallow water and tidal flat areas of the Ariake Sea, Japan: Environmental fate of perfluorooctane sulfonate in aquatic ecosystems. Environ. Sci. Technol..

[59] Houde M., De Silva A.O., Muir D.C., Letcher R.J. (2011). Monitoring of perfluorinated compounds in aquatic biota: An updated review. Environ. Sci. Technol..

[60] Zhao L., Zhu L., Yang L., Liu Z., Zhang Y. (2012). Distribution and desorption of perfluorinated compounds in fractionated sediments. Chemosphere.

[61] Casal P., Gonzalez-Gaya B., Zhang Y., Reardon A.J., Martin J.W., Jimenez B., Dachs J. (2017). Accumulation of Perfluoroalkylated Substances in Oceanic Plankton. Environ. Sci. Technol..

[62] Liu Y., Ruan T., Lin Y., Liu A., Yu M., Liu R., Meng M., Wang Y., Liu J., Jiang G. (2017). Chlorinated Polyfluoroalkyl Ether Sulfonic Acids in Marine Organisms from Bohai Sea, China: Occurrence, Temporal Variations, and Trophic Transfer Behavior. Environ. Sci. Technol..

[63] Chen S., Yan M., Chen Y., Zhou Y.Q., Li Z.C., Pang Y. (2022). Perfluoroalkyl substances in the surface water and fishes in Chaohu Lake, China. Environ. Sci. Pollut. Res..

[64] Liu S., Wang T., Xue K. (2017). Occurrence and human health risk of PFASs in fishes from drinking water sources of Beijing. Asian J. Ecotoxicol..

[65] Chen M., Zhu L., Wang Q., Shan G.Q. (2021). Tissue distribution and bioaccumulation of legacy and emerging per-and polyfluoroalkyl substances (PFASs) in edible fishes from Taihu Lake, China. Environ. Pollut..

[66] Wang S., Cai Y., Ma L., Lin X., Li Q., Li Y., Wang X. (2022). Perfluoroalkyl substances in water, sediment, and fish from a subtropical river of China: Environmental behaviors and potential risk. Chemosphere.

[67] Zhang Y., Liu X., Yu L., Hua Z.L., Zhao L., Xue H.Q., Tong X.N. (2022). Perfluoroalkyl acids in representative edible aquatic species from the lower Yangtze River: Occurrence, distribution, sources, and health risk. J. Environ. Manag..

[68] Li Y., Yao J., Pan Y., Dai J.Y., Tang J.H. (2023). Trophic behaviors of PFOA and its alternatives perfluoroalkyl ether carboxylic acids (PFECAs) in a coastal food web. J. Hazard. Mater..

[69] Martin J.W., Whittle D.M., Muir D.C.G., Mabury S.A. (2004). Perfluoroalkyl Contaminants in a Food Web from Lake Ontario. Environ. Sci. Technol..

[70] Gao K., Miao X., Fu J., Chen Y., Li H.J., Pan W.X., Fu J.J., Zhang Q.H., Zhang A.Q., Jiang G.B. (2020). Occurrence and trophic transfer of per- and polyfluoroalkyl substances in an Antarctic ecosystem. Environ. Pollut..

[71] Zhang Z., Peng H., Wan Y., Hu J.Y. (2015). Isomer-specific trophic transfer of perfluorocarboxylic acids in the marine food web of Liaodong Bay, North China. Environ. Sci. Technol..

[72] Zhong T., Lin T., Zhang X., Jiang F.C., Chen H. (2023). Impact of biological activated carbon filtration and backwashing on the behaviour of PFASs in drinking water treatment plants. J. Hazard. Mater..

[73] Chen R., Huang X., Li G., Yu Y., Shi B.Y. (2022). Performance of in-service granular activated carbon for perfluoroalkyl substances removal under changing water quality conditions. Sci. Total Environ..

[74] Liu L., Liu Y., Gao B., Ji R., Li C.L., Wang S.S. (2019). Removal of perfluorooctanoic acid (PFOA) and perfluorooctane sulfonate (PFOS) from water by carbonaceous nanomaterials: A review. Crit. Rev. Environ. Sci. Technol..

[75] Liu Z., Pan C., Peng F., Hu J.J., Tan H.M., Zhu R.G., Liang H., Yu K.F. (2024). Rapid adsorptive removal of emerging and legacy per- and polyfluoroalkyl substances (PFASs) from water using zinc chloride-modified litchi seed-derived biochar. Bioresour. Technol..

[76] Cheng L., Detlef K. (2024). Removal of Per- and Polyfluoroalkyl substances by anion exchange resins: Scale-up of rapid small-scale column test data. Water Res..

[77] Parvin S., Hara H., Kanai Y., Yamasaki A., Adachi T., Sorn S., Honda R., Yamamura H. (2023). Important properties of anion exchange resins for efficient removal of PFOS and PFOA from groundwater. Chemosphere.

[78] Steve W., John B., Brandon N. (2017). Ion exchange resin for PFAS removal and pilot test comparison to GAC. Remediat. J..

[79] Deng S., Nie Y., Du Z., Huang Q., Meng P.P., Wang B., Huang J., Yu G. (2015). Enhanced adsorption of perfluorooctane sulfonate and perfluorooctanoate by bamboo-derived granular activated carbon. J. Hazard. Mater..

[80] Abu Z., Bikash B., Rominder P. (2017). Environmentally Friendly β-Cyclodextrin–Ionic Liquid Polyurethane-Modified Magnetic Sorbent for the Removal of PFOA, PFOS, and Cr(VI) from Water. ACS Sustain. Chem. Eng..

[81] Deng S., Bei Y., Lu X., Du Z.W., Wang B., Wang Y.J., Huang J., Yu G. (2015). Effect of co-existing organic compounds on adsorption of perfluorinated compounds onto carbon nanotubes. J. Hazard. Mater..

[82] Deng S., Yu Q., Huang J., Yu G. (2010). Removal of perfluorooctane sulfonate from wastewater by anion exchange resins: Effects of resin properties and solution chemistry. Water Res..

[83] Alessandro Z., Lino C., Luigi F., Fant M., Chiorboli A. (2016). Use of strong anion exchange resins for the removal of perfluoroalkylated substances from contaminated drinking water in batch and continuous pilot plants. Water Res..

[84] Brian C., Thomas F., David G., Smith S.J., Abulikemu G., Kleiner E.J., Pressman J.G. (2019). Occurrence of per- and polyfluoroalkyl substances (PFAS) in source water and their treatment in drinking water. Crit. Rev. Environ. Sci. Technol..

[85] Aron M., Christopher B., Timothy J. (2024). Rejection of PFAS and priority co-contaminants in semiconductor fabrication wastewater by nanofiltration membranes. Water Res..

[86] Ma Q., Lei Q., Liu F., Song Z.M., Khusid B., Zhang W. (2024). Evaluation of commercial nanofiltration and reverse osmosis membrane filtration to remove per-and polyfluoroalkyl substances (PFAS): Effects of transmembrane pressures and water matrices. Water Environ. Res..

[87] Mustafa N., Sama A., Sibel B., Koseoglu-Imer D.Y., Dumée L.F., Shirazi M.M.A. (2024). Progress on remediation of per- and polyfluoroalkyl substances (PFAS) from water and wastewater using membrane technologies: A review. J. Water Process Eng..

[88] Ilango A.K., Arathala P., Musah R.A., Liang Y. (2024). Experimental and density functional theory investigation of surface-modified biopolymer for improved adsorption of mixtures of per- and polyfluoroalkyl substances in water. Water Res..

[89] Fu K., Huang J., Luo F., Fang Z.Y., Yu D.Y., Zhang X.L., Wang D.W., Xing M.Y., Luo J.M. (2024). Understanding the Selective Removal of Perfluoroalkyl and Polyfluoroalkyl Substances via Fluorine–Fluorine Interactions: A Critical Review. Environ. Sci. Technol..

[90] Liang G., Xu S., Han Z., Yang Y.H., Wang K.Y., Huang Z.H., Rushlow J., Cai P.Y., Samorì P., Zhou H.C. (2024). Exceptionally High Perfluorooctanoic Acid Uptake in Water by a Zirconium-Based Metal–Organic Framework through Synergistic Chemical and Physical Adsorption. J. Am. Chem. Soc..

[91] Amin Z., Ahmadreza K., Amin Z., Khazdooz L., Amirjalayer S., Auras F., Abbaspourrad A. (2024). Viologen-Derived Covalent Organic Frameworks: Advancing PFAS Removal Technology with High Adsorption Capacity. Small.

[92] Guo H., Hu T., Yang X., Liu Z.Y., Cui Q.Q., Qu C.C., Guo F.Y., Sweetman A.J., Hou J.T., Tan W.F. (2024). Roles of varying carbon chains and functional groups of legacy and emerging per-/polyfluoroalkyl substances in adsorption on metal-organic framework: Insights into mechanism and adsorption prediction. Environ. Res..

[93] Wang P., An G., Irene C., Hassard F., Moreno P.C., Sakar H., Jodkowska M., Wang D.S., Jefferson B., Chu W.H. (2025). Removal of perfluorooctanoic acid (PFOA) and perfluorooctanesulfonic acid (PFOS) by coagulation: Influence of coagulant and dosing conditions. Sep. Purif. Technol..

[94] Amith S., Zhang Y., Jonathan L., Venkatasan A.K. (2024). Surfactant-enhanced coagulation and flocculation improves the removal of perfluoroalkyl substances from surface water. Environ. Sci. Adv..

[95] Michel H., Thomas M., Mona C., Hale S.E., Arp H.P.H. (2024). Per- and polyfluoroalkyl substance (PFAS) removal from soil washing water by coagulation and flocculation. Water Res..

[96] Debanjali D., Tajamul S., Shamik C., Dubey B.K., Sen R. (2024). Progress and perspectives on carbon-based materials for adsorptive removal and photocatalytic degradation of perfluoroalkyl and polyfluoroalkyl substances (PFAS). Chemosphere.

[97] Javier L., Carla S., Rosa M., Rodil R., Quintana J.B., Gäbler J., Schäfer L., Moreira F.C., Vilar V.J.P. (2024). Insights into the application of the anodic oxidation process for the removal of per- and polyfluoroalkyl substances (PFAS) in water matrices. Chem. Eng. J..

[98] Kaushik L., Arjun K. (2024). Effect of chain length, electrolyte composition and aerosolization on the removal of per- and polyfluoroalkyl substances during electrochemical oxidation. Environ. Sci. Water Res. Technol..

[99] Sivasai P., Narasamma N. (2024). Advanced insights into sustainable electrooxidation technique and futuristic strategies: Multifaceted approach for PFAS degradation. J. Environ. Chem. Eng..

[100] Ma H., Kang Y., Li M., Dong J.H., Wang Y.Q., Xiao J.Q., Guo Z.Z. (2023). Enhancement of perfluorooctanoic acid and perfluorooctane sulphonic acid removal in constructed wetland using iron mineral: Performance and mechanisms. J. Hazard. Mater..

[101] Pinelopi S., Gabriela D., Pablo C., Coulon F., Lyu T. (2024). Constructed wetlands as nature-based solutions in managing per-and poly-fluoroalkyl substances (PFAS): Evidence, mechanisms, and modelling. Sci. Total Environ..

[102] Rao D., Liu J. (2024). Photochemical PFAS Degradation in Ion Exchange Resin Regeneration Brine: Effects of Water Matrix Components and Technical Solutions. ChemRxiv.

[103] Leen D., George M., Lilian M., Zayyat R.M. (2024). A review on the occurrence of per- and polyfluoroalkyl substances in the aquatic environment and treatment trends for their removal. J. Environ. Chem. Eng..

